# Experimental Validation of Comprehensive Calculation for High-Resolution Linear MALDI-TOF Mass Spectrometry

**DOI:** 10.1021/jasms.4c00018

**Published:** 2024-04-18

**Authors:** Yi-Hong Cai, Chia-Chen Wang, Chih-Hao Hsiao, Yi-Sheng Wang

**Affiliations:** † Genomics Research Center, Academia Sinica, Taipei 115, Taiwan ROC; ‡ Instrumentation Center, National Taiwan Normal University, Taipei 106, Taiwan ROC

## Abstract

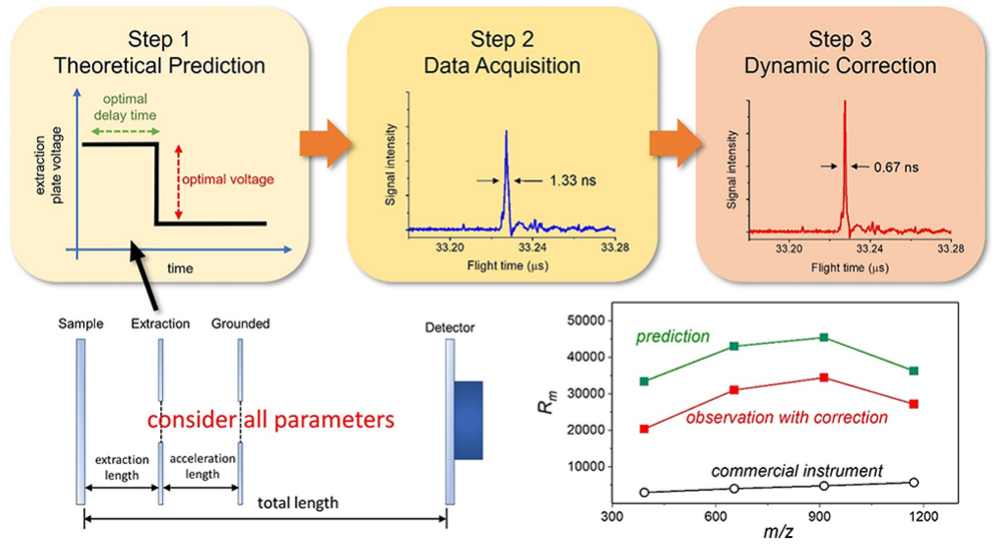

This work discusses the effectiveness of the previously developed comprehensive calculation model to optimize linear MALDI-TOF mass spectrometers. The model couples space- and velocity-focusing to precisely analyze the flight-time distribution of ions and predict optimal experimental parameters for the highest mass resolving power. Experimental validation was conducted using a laboratory-made instrument to analyze CsI_3_ and angiotensin I ions in low to medium *m*/*z* range. The results indicate that the predicted optimal extraction voltage and delay were reasonably accurate and effective. In the low *m*/*z* range, the peak width obtained using optimal parameters reached the sub nanosecond range, corresponding to a mass resolving power of 10 000–17 000, or 20 000–34 000 if shot-to-shot random fluctuations were minimized by the dynamic data correction method. The observed optimal mass resolving power in the current experiment is 4.8–7.8 times that of commercial instruments. Practical limitations resulting in the gap between the observed and theoretical ultimate mass resolving power are discussed.

## 1 Introduction

Improving the performance of linear time-of-flight (TOF) mass spectrometers is a challenging subject that receives little current attention.^1−4^ Although linear TOFMS offers much lower mass resolving power (*R*_m_) than other advanced variants (i.e., reflectron, orthogonal acceleration, multireflection, etc.) of TOFMS or Fourier-Transform MS,^5−8^ it is advantageous for analysis across a wide *m*/*z* range.^9^ For example, linear TOFMS combining matrix-assisted laser desorption/ionization (MALDI) ion source is the benchmark for the identification of bacterial strains and polymers^10−13^ with such samples being typically polydisperse and structurally complicated. The mass range of such samples are beyond the working range of most high-resolution mass spectrometry. We have demonstrated that a miniature linear TOF mass spectrometer optimized by the calculation model offered higher sensitivity and resolution for polymers and bacterial strains than a regular-sized commercial instrument.^14^ Therefore, improving the performance of linear TOFMS, including its sensitivity and *R*_m_, in an efficient way is necessary for expanding its application to more detailed and unsolved analytical problems. Examples include distinguishing antibiotic resistant bacterial species^15^ or differentiating linear and cyclic polymers.^16^ To achieve this goal, a rational theoretical basis or tool for instrument development and optimization is necessary.

Although linear TOF mass spectrometers have a simple configuration, they offer unique features when coupled with a MALDI ion source. First, the simple configuration preserves TOFMS’s extremely high sensitivity since ions need not travel in a complicated trajectory.^17−20^ Second, combined MALDI-TOF mass spectra are comprehensible since MALDI typically produces singly charged ions. In contrast, present high-resolution MS typically uses an electrospray ionization (ESI) source that generates multiply charged molecules,^9,21,22^ resulting in complicated spectra due to overlapping *m*/*z* envelopes between different species and making the interpretation of mass spectra inconvenient.^23−25^ The drawback of linear MALDI-TOFMS is that a rational principle for instrument optimization has been unavailable in the past.^17^

A comprehensive calculation model to accurately predict the *R*_m_ of linear MALDI-TOF mass spectrometers was previously developed in our laboratory.^26−29^ It addresses the decline in *R*_m_ due to the initial velocity spread of ions.^30−32^ The model revealed for the first time how most instrumental parameters affect the resultant *R*_m_ more prominently than expected.^4,26^ However, our analysis showed that the consensus toward increasing flight distance being capable for increasing *R*_m_ was only conditionally valid. Instead, the *R*_m_ of an instrument is sensitive to the dimensions of every ion manipulation region, voltages, and extraction delay. In commercial linear TOF spectrometers, the parameters are usually set empirically, with *R*_m_ always limited to within 10 000. Our new calculation model can predict suitable parameters to considerably improve the *R*_m_ of existing instruments.^27,29^

The objective of this study is to verify the effectiveness of the calculation model. We apply the model to optimize a laboratory-made linear MALDI-TOF mass spectrometer that focuses on the low *m*/*z* region. Two standard substances were used to systematically evaluate the performance, including cesium triiodide and angiotensin I. The change in *R*_m_ in response to the changes in extraction voltage, extraction delay, and ion mass was carefully analyzed. The adjustable range for the extraction voltage was constrained by the electronic circuit system, while the optimal extraction delay was determined theoretically. The experimental results indicate that the predicted parameters were accurate, and the observed *R*_m_ could achieve about 4.8 to 7.8 times that of commercial instruments, but they were still 30–40% lower than that of predicted values. The reasons and practical limitations for the deviation between the theoretical and experimental values are discussed.

## 2 Experimental Section

### Instrument Design

The linear TOF mass spectrometer was manufactured in-house, comprising a Wiley–McLaren-type^1^ two-stage extraction MALDI ion source and a field-free flight tube. The ion source encompasses an extraction and an acceleration region with lengths of 8 (*s*_0_) and 10 (*d*) mm, respectively. The total length of the instrument (*L*) is 3236 mm. The instrument design is similar to commercial instruments, except the length of its flight tube is longer. Figure S1 in the Supporting Information (SI) shows the schematics of the instrument. Ions are produced by utilizing a Nd:YLF laser with a wavelength of 349 nm (Explorer 349 nm ICT-349-120-E, MKS Instruments, Stahnsdorf, Germany) and detected using an ultrafast microchannel plate detector (F9890-31/-32, Hamamatsu Photonics K.K. Shizuoka, Japan). The detector offers a response time or detection limit of roughly 0.5 ns, making it one of the fastest products available on the market. To evaluate the instrument performance, the same samples were also analyzed using the linear mode of a commercial high-performance MALDI-TOF mass spectrometer (Ultraflex II TOF/TOF, Bruker Daltonics, Billerica, MA, U.S.A.).

### Sample Preparation

Cesium triiodide (CsI_3_, 99.9%), angiotensin I human acetate salt hydrate (90%), and α-cyano-4-hydroxycinnamic acid (CHCA) were purchased from Sigma-Aldrich (St. Louis, MO, U.S.A.). Organic solvents used in sample preparation, including acetonitrile (99.9%) and ethanol (99.9%), were sourced from J. T. Baker Avantor (Radnor, PA, U.S.A.). Distilled deionized water used in the experiment (with a resistivity of 18.2 MΩ-cm) was produced by the Merck milli-Q purification system (Darmstadt, Germany).

The CsI_3_ analyte solution was prepared using 50% ethanol with a concentration of 0.1 mmol/mL. One microliter of the analyte solution was directly deposited onto the sample plate and dried in a vacuum chamber before analysis. The preparation process did not involve any matrix mixing, thereby avoiding interference of mass spectra from matrix signals. The angiotensin I analyte solution was dissolved in distilled deionized water at a concentration of 10 nmol/mL. The CHCA solution was prepared using 50% aqueous acetonitrile solution, with a concentration of 0.1 mmol/mL. The CHCA matrix and angiotensin I solutions were premixed in a final matrix-to-analyte molar ratio of 100:1. Finally, one microliter of the premixed solution was deposited onto the sample plate and dried in the vacuum chamber.

### Comprehensive Calculation and Instrument Optimization

The calculation was conducted using Mathematica 11.3 (Wolfram Research, Champaign, IL, U.S.A.) based on the comprehensive calculation model developed previously.^26−29^ It can complete the prediction of the best parameters and resultant *R*_m_ in roughly 3–5 s. In this study, the sample-plate voltage was fixed at +20 000 V both theoretically and experimentally. Since the instrument dimensions and sample-plate voltages were fixed, a suitable extraction voltage (*V*_s_) and delay (τ) for ions of different *m*/*z* could be calculated. The ions analyzed in the current work include *m*/*z* 392, 652, 912, and 1172, corresponding to (CsI)_*n*_Cs^+^ clusters with respective n of 1 to 4, and *m*/*z* 1297, corresponding to protonated angiotensin I. The prediction results served as starting parameters for fine adjustments during measurement. Unless mentioned otherwise, every CsI_3_ spectrum integrated 20 single acquisition events, and for angiotensin I, 100 single events were used. The experiments were repeated at least three times to ensure reproducibility.

*R*_m_ was calculated using the ions’ flight time (*t*) and flight-time spread (Δ*t*), i.e. *R*_m_ = *t*/2Δ*t*. In the prediction, ions within the full-width at half-maximum (fwhm) of the Maxwell–Boltzmann initial velocity distribution were used to calculate flight time distributions (FTD). The *t* in the predicted *R*_m_ is the average flight time of the first and last ions arriving at the detector, whereas the Δ*t* is the time difference between the two ions. In experimental observations, the flight time of the peak maximum and the fwhm of the peak were used to calculate the experimental *R*_m_.

### Peak Alignment Method

A dynamic data correction (DDC) software to minimize peak broadening effect due to shot-to-shot random errors was utilized to improve the experimental *R*_m_.^33^ The software was developed using the C++ programming language and operated on the Microsoft Windows 11 system. It compared and corrected peak positions in every single acquisition event, and integrated all corrected spectra to generate the final spectrum. The tolerances of peak width reported in this work represent the standard deviation. The calibration range was 1–2 standard deviations with respect to the central flight time, which was 1–2 ns for ions of *m*/*z* 392. The observed spectral features were used without flight-time/mass calibration, and the variation across different experiments was roughly 0.025%.

## 3 Results and Discussion

### 3.1 Accuracy of Theoretical Calculation

#### 3.1.1 Flight Time

The accuracy and reliability of the calculation was first checked by comparing the predicted and observed *t* of representative ions. Figure S2 shows the result of CsI_3_ cluster ions, in which the predicted and observed *t* agree with each other when optimal parameters were employed. The differences between the prediction and observation were less than 0.6%, which is accurate enough for critical evaluation of the model. Notably, since the calculation results are reliable and they facilitate acquisition of very high-resolution spectra on the instrument, we were able to observe changes in *t* due to tiny changes in τ, voltages, and even random fluctuation of spectral features. To the best of our knowledge, random fluctuations were undetectable in the past mainly because the minimum peak width observed using conventional instruments were above 3 ns, making it difficult to resolve adjacent peaks or peak fluctuations within this time. In fact, this is the first work that demonstrates the effectiveness of the theoretical model on finding the limit of such an instrument.

#### 3.1.2 Extraction Delay

The accuracy of τ is selected to further evaluate the effectiveness of the calculation model. Although the appropriate τ is very sensitive to other experimental parameters (i.e., ion mass, instrument dimension details, voltages), the calculations in this work were simplified since instrument dimensions were fixed. This optimization scenario is similar to the case in commercial instruments, in which the adjustable parameters are voltages and τ.

To achieve a value of *R*_m_ close to the predicted ultimate value, τ and the *V*_s_ need to be within a certain range that approximate their best values. Taking (CsI)Cs^+^ (*m*/*z* 392) as an example, based on calculations, changing the *V*_s_ to within 778–993 V allows for the observed *R*_m_ to reach greater than 99.7% of the ultimate *R*_m_ if its respective optimal τ is utilized. Alternatively, when fixing the voltage to a value within the optimal range, such as 860 V, for τ to achieve the same 99.7% of the ultimate *R*_m_, it must be between 960–990 ns. However, due to the lack of a reliable optimization principle, most MS users only adjust τ and keep voltage constant.

**Figure 1:**
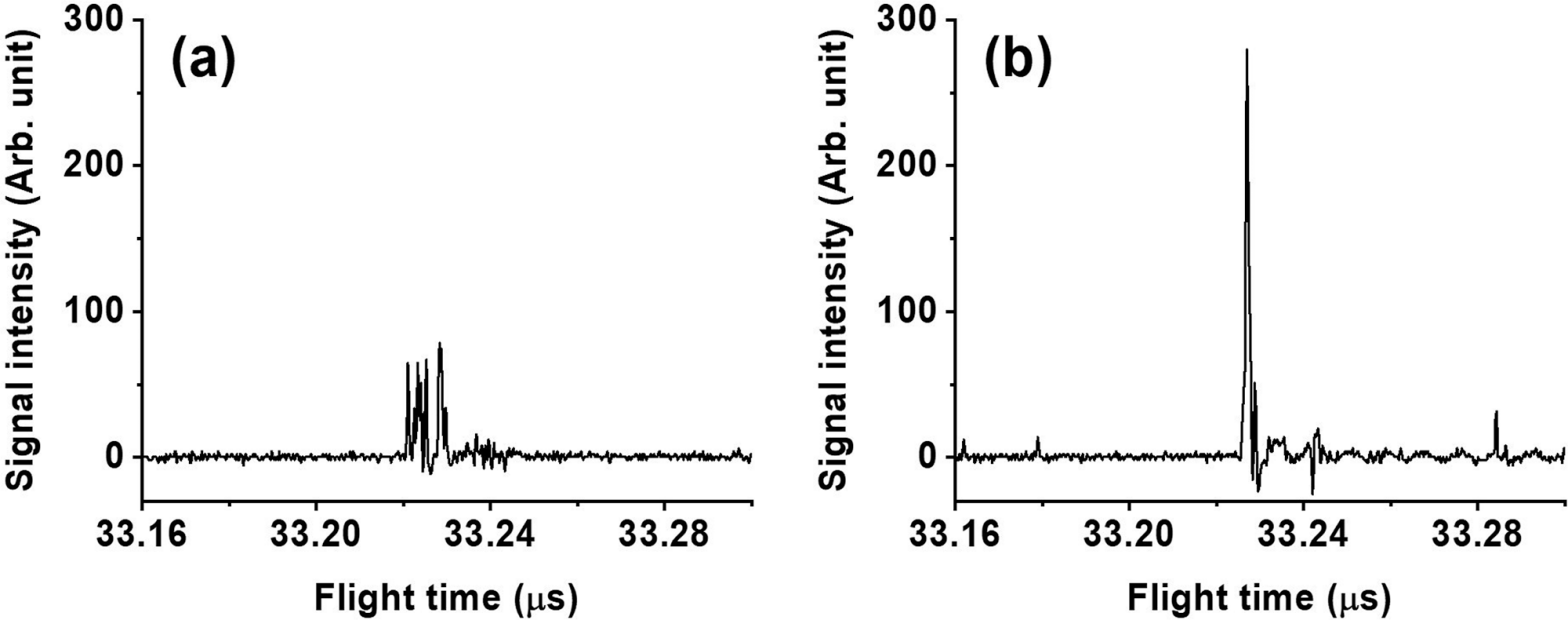
Mass spectra of ions of *m*/*z* 392 with one laser shot obtained using (a) nonoptimized (310 ns) and (b) optimized (970 ns) extraction delays.

The experimental results agree with the calculation in the case of *m*/*z* 392. For instance, the calculation indicated that an *V*_s_ of 860 V and a τ of 975 ns was able to achieve a result very close to the ultimate *R*_m_. Figures 1a and 1b compare the single-shot mass spectrum of (CsI)Cs^+^ respectively obtained using nonoptimized (310 ns) and optimized (970 ns) delays while keeping the *V*_s_ at 860 V. The results show multiple features distributed across a range of about 7.5 ns when using the nonoptimized delay (Figure 1a), whereas there was only a single feature with a peak width of 0.7 ± 0.1 ns when τ was 970 ns (Figure 1b). Since the response limit of the detector is 0.5 ns, the peak width obtained using 970 ns was very close to the limit, indicating almost all ions arrived at the detector at the same time. Notably, the individual features in Figure 1a were attributed to a single ion based on peak shape and intensity. It is evident that the optimization method introduced herein can effectively focus ion packets of single laser shots in the low *m*/*z* range. When nonoptimized τs were employed, the ions were not properly focused, and they exhibited different arrival times. Such inappropriate parameters deteriorated the spectral quality.

**Figure 2:**
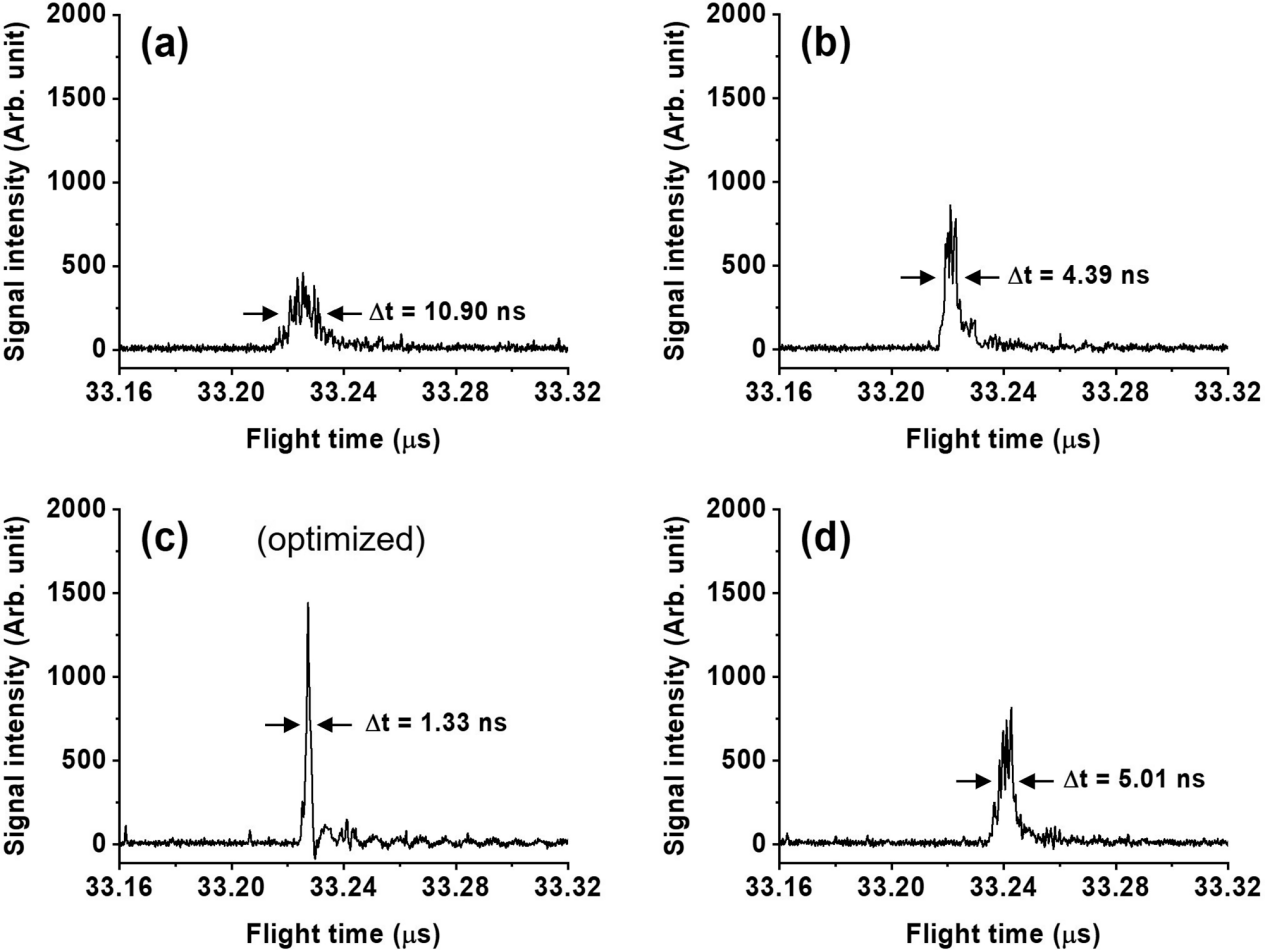
Mass spectra of CsICs^+^ (*m*/*z* = 392) obtained with 20 laser shots when using a delay of (a) 310 ns, (b) 610 ns, (c) 970 ns, and (d) 1510 ns.

The average peak shapes obtained by accumulating multiple laser shots clearly show how the parameters affect observed mass spectra. The spectral features of the same ion observed experimentally using 20 laser shots with 860 V and with τ values of 310, 610, 970, and 1510 ns are displayed in Figures 2a–d. When using a 310 ns delay (Figure 2a), the spectrum showed a feature with a fwhm of 10.9 ns and an arbitrary intensity of roughly 300. The spectral feature has an *R*_m_ of 1523. When increasing τ to 610 ns (Figure 2b), the peak width became 4.3 ns and the intensity was roughly 650. This corresponds to an *R*_m_ of 3781. When τ was 970 ns (Figure 2c), the peak reached its minimum width of 1.3 ns and an intensity of 1441, corresponding to an *R*_m_ of 12 464. The observed best τ was only roughly 0.6% less than the prediction, indicating the calculation model is highly accurate in this *m*/*z* range. When further increasing τ to 1510 ns (Figure 2d), the peak width became 5.0 ns and intensity was roughly 680. The results clearly show that *R*_m_ is highly sensitive to τ, and the signal intensity considerably increases when the best τ is employed.

A more sophisticated analysis was performed by fixing the *V*_s_ at 930 V and adjusting τ more finely. In this case, according to the prediction, *R*_m_ should reach its maximum value when τ is approximately 736 ns and is reduced by roughly 20% when τ deviates by 20 ns, as shown in Figure S4 (see SI). Experimental measurements showed that the best *R*_m_ was obtained when τ was roughly 740 ns, which matched nicely with the prediction. The only difference between the experiment and the prediction was that the experimental *R*_m_ was not as sensitive to τ as expected. Observation shows that it reduced by ∼20% when τ changed by 80 ns. However, when τ shifted from its best value, signal intensity did reduce constantly. The peaks became difficult to analyze when τ shifted beyond 80 ns. The result suggests that τ still needs to be precisely determined to obtain appropriate sensitivity and resolution. In summary, the calculated value of τ is accurate and serves as an effective reference for gaining optimal performance, greatly accelerating the optimization process.

### 3.2 Reduction of Random Errors

When pushing an instrument into the very high *R*_m_ region, the peak width may become narrow enough to make shot-to-shot signal fluctuation discernible. The magnitude of random fluctuation/errors in the spectral feature at around *m*/*z* 392 for this instrument, or the standard deviation of the peak position, was roughly 1 ns (see Figure S3). Therefore, if the spectral features of every single laser shot are narrower than this value, the impact of random fluctuation on spectra quality after accumulating multiple laser shots is not negligible. Such fluctuation results in peak broadening in the final spectra. Figures 1b and 2c represent such a case since peak width with a single laser shot was about 0.7 ns, whereas at an average of 20 laser shots, the peak width became 1.3 ns. The error in this instrument mainly arises from fluctuations in the experimental environment, including the stability of the electric system. In conventional instruments, the peak width of spectral features always exceeds the detector’s response limit, so the influence of random error on *R*_m_ is not pronounced.

**Figure 3:**
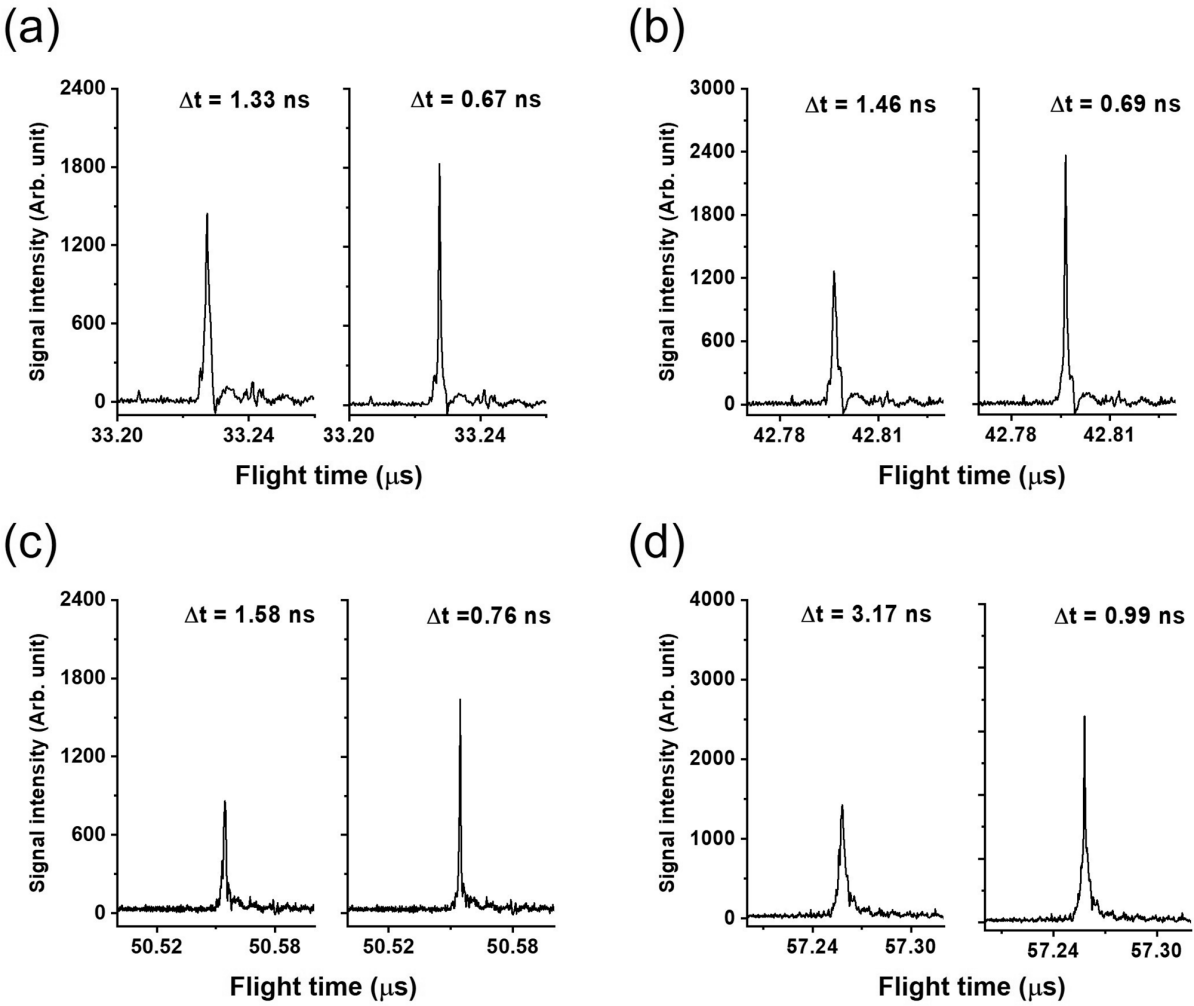
Mass spectra of *m*/*z* (a) 392, (b) 652, (c) 912, and (d) 1172 with 20 laser shots after with (left) and without (right) dynamic data correction.

In order to minimize the impact of random errors on resultant peak shapes, individual mass spectra were processed using the DDC method. Figures 3a–d show the mass spectra of various CsI_3_ cluster ions accumulating 20 laser shots using optimal experimental parameters (see detailed discussion in Section 3.3). For instance, Figure 3a reveals that the peak width of the *m*/*z* 392 ions reduced from 1.33 ns to about 0.7 ns after DDC, corresponding to a resultant *R*_m_ of approximately 24 682, which is almost double the performance. The magnitude of improvements observed for *m*/*z* 392–912 ions were very similar. However, it needs to be emphasized that the DDC method should be used when the *V*_s_ and τ are well optimized. It cannot be used for spectra with poor resolution and signal intensity since it may distort the peak shape and make data interpretation problematic.

### 3.3 Experimental Validation of Instrument Performance

Although the experimental data demonstrated above show that the calculation was effective for finding suitable ranges of these parameters in the low *m*/*z* range, the question remains whether such calculations can be extended to higher *m*/*z* ranges and the instrument can actually produce the same spectral quality as predicted. For CsI_3_ cluster ions in the *m*/*z* range: 392, 652, 912, and 1172 as shown in Figures 3a–d, the predicted optimal *V*_s_ falls within 800–1200 V. On the basis of calculation, the respective *V*_s_/τ values are 860 V/955 ns, 930 V/927 ns, 990 V/910 ns, and 1040 V/859 ns. The voltages were confirmed experimentally to be suitable in systematic measurements that underwent fine-tuning processes, so the respective voltages were utilized and fixed in subsequent measurements, but τ was changed arbitrarily. In every case, the spectrum corrected using the DDC method was demonstrated for comparison. The related experimental parameters and results are summarized in Table 1.

**Table 1:** Optimal Parameters and Results Obtained by Prediction and Experimental Observation in Figure 3^a^

		prediction			experiment		
*m*/*z*	*V*_s_ (V)	τ (ns)	Δ*t* (ns)	*R*_m_	τ (ns)	Δ*t* (ns)	*R*_m_
392	860	975	0.24	33 407	970	0.67	24 682
652	930	955	0.38	43 022	870	0.69	31 001
912	990	927	0.56	45 421	810	0.76	33 439
1172	1040	910	0.78	36 292	790	0.99	28 712
1297	1080	859	0.90	33 750	826	1.20	25 086

a The experimental data are corrected by dynamic data correction.

The improvement in spectral quality after careful optimization for most CsI_3_ cluster ions was similar to that of the (CsI)Cs^+^ ion discussed above. The optimal τ observed experimentally for *m*/*z* 652 ion ((CsI)_2_Cs^+^) was 870 ns (Figure 3b), which was roughly 9% lower than the prediction (955 ns). The central flight time of this ion was roughly 42.80 μs, and the best peak width was approximately 0.69 ns, corresponding to an *R*_m_ of 31 001. The observed *R*_m_ was approximately 35% lower than the prediction. This was considered the detector’s response limit. In the case of *m*/*z* 912 (Figure 3c), experimental results show that the smallest peak width was approximately 0.76 ns, corresponding to an *R*_m_ of around 33 439, obtained using τ of 810 ns. The observed τ was about 13% lower than the prediction (927 ns), and the observed *R*_m_ was roughly 25% lower than the prediction. In the case of *m*/*z* 1172 (Figure 3d), the observed optimal τ was about 790 ns, which was about 8% lower than the prediction. The observed peak width was about 0.99 ns, corresponding to an *R*_m_ of approximately 28 712, which was 28% lower than prediction.

**Figure 4:**
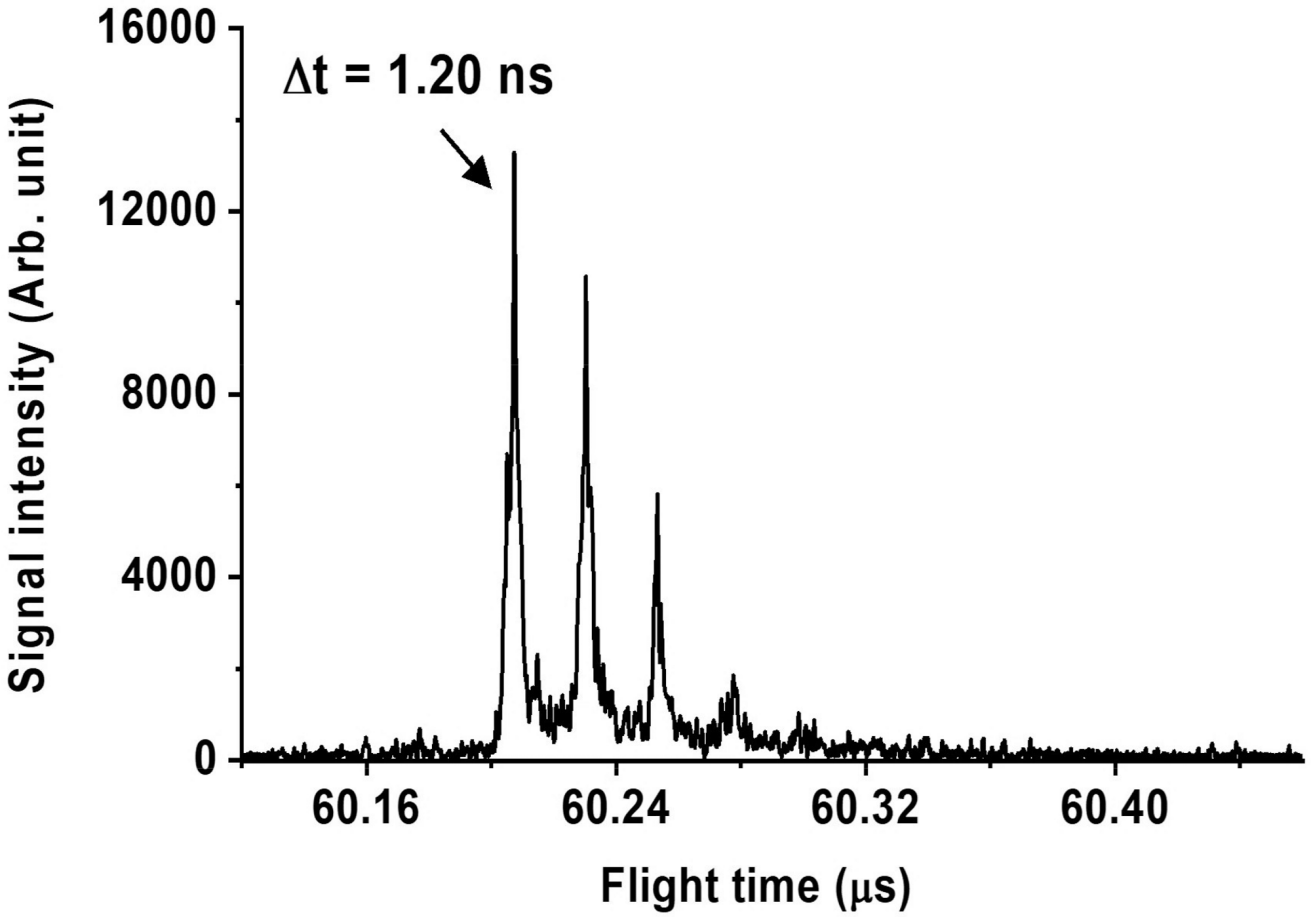
Mass spectrum of protonated angiotensin I with 100 laser shots after dynamic data correction.

The same result was obtained with angiotensin I. Although the low *m*/*z* range of angiotensin I spectra were complicated by the presence of MALDI matrix signals, the feature of angiotensin I ion was reasonably intense and clean. Figure 4 shows the main features of protonated angiotensin I after optimization and accumulation of 100 laser shots, in which the isotopologues can be distinguished unambiguously. The achieved peak width was roughly 1.2 ns after DDC, corresponding to an *R*_m_ of 25 086. This *R*_m_ was 3–4 times higher than that of standard commercial mass spectrometers. Without optimization, the instrument was unable to resolve the isotopologues due to the poor *R*_m_.

The data confirms that the calculation model was helpful for predicting optimal experimental parameters and results. The predicted τ values were accurate enough to be within 13%, efficiently minimizing the time for random trials. The calculation was also helpful for predicting the trends in resultant spectral quality. For example, the prediction suggested that the peak width for ions of *m*/*z* 392, 652, and 912 will be similar when considering the detector’s response limit, and the peak width of *m*/*z* 1172 ion is predicted to be roughly 39% more than the *m*/*z* 912 ion. Experimental observations resembled the result: the difference in peak width of ions of *m*/*z* 392, 652, and 912 was less than 5%, and the peak width of *m*/*z* 1172 ion was 41% more than the *m*/*z* 912 ion. The observed peak width of angiotensin I ion was 21% wider than the *m*/*z* 912 ion, whereas the calculation predicted it to be roughly 15%.

However, in this study, the smallest peak width obtained experimentally lies between 0.6–0.8 ns. Such values are 20–60% greater than the detector’s response limit. As mentioned in our previous studies, there are practical limitations to be overcome in reality, such as the inhomogeneity of the electric field inside the ionization source and the finite rising time of high voltage for delayed extraction. These additional factors might increase the minimum Δ*t*, but obviously they are not discernible in conventional instruments and can be disregarded. Such limitations are important only when the instrument’s performance has been pushed to its extreme.

### 3.4 Comparison between Laboratory-Made and Commercial Mass Spectrometers

It needs to be emphasized that the calculation model can be applied to other linear TOF mass spectrometers that possess the same configuration. Unfortunately, many commercial products use different ion source designs and they cannot directly benefit from the calculation model. In such cases, a scaling formula probably needs to be incorporated. The commercial instrument utilized in the current work represents such a case since it consists of a gridless curved extraction electrode for the ion source. Therefore, the optimization of this commercial instrument was solely based on experience, including the modulation of both *V*_s_ and τ. Both the experimental results and the specifications of this instrument indicated that peak *R*_m_ values are between 2500 and 5500.

**Figure 5:**
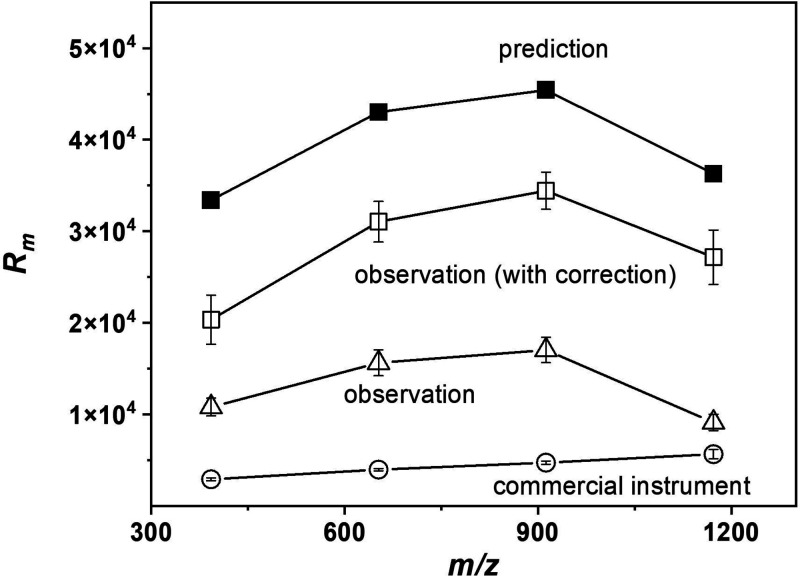
Highest *R*_m_ for *m*/*z* 392, 652, 912, and 1172 achieved in the calculation, the laboratory-made instrument, and the commercial instrument.

Figure 5 compares the best *R*_m_ of ions obtained using laboratory-made and commercial instruments. The results show that the highest *R*_m_ value for the *m*/*z* 392 ion in the commercial instrument was approximately 2936, while the *R*_m_ obtained after DDC in our instrument was 6.9 times higher. For the *m*/*z* 652 ion, the *R*_m_ of the commercial instrument was 3984, while the *R*_m_ obtained after DDC in our instrument was about 7.8 times higher. When *m*/*z* increased to 912, the *R*_m_ in the commercial instrument was 4753, while the *R*_m_ obtained in our instrument was about 7.2 times higher. For the *m*/*z* 1172 ion, the highest *R*_m_ in the commercial instrument was 5674, while the *R*_m_ in our instrument was about 4.8 times higher. One may argue that the 4.8–7.8 times enhancement in the laboratory-made instrument was due to the longer flight tube. Our estimations suggest that reducing the instrument length to that of the commercial instrument would still result in improved *R*_m_ in the range of 3.0–4.8 times.

However, the theoretical prediction for the *R*_m_ of the laboratory-made instrument is approximately: 33 407, 43 022, 45 421, and 36 292 for ions of *m*/*z* of: 392, 652, 912, and 1172, respectively. The observed *R*_m_ values for those ions are respectively: 10 818, 15 652, 17 047, and 9141 before DDC, and 20 366, 31 052, 34 434, and 27 173 after DDC. Although the results indicate that improvements attributable to the DDC are in the range of about 1.8–3.0 times, there are still respective anomolies of: 39%, 28%, 24%, and 25% less than predictions. Such discrepancies are attributed to imperfections in the instrument (e.g., fringe electric fields and slow voltage pulses), as discussed in Section 3.3.

## 4 Conclusions

The optimal experimental parameters predicted by the comprehensive calculation method conform well with the actual experimental results. The predicted values serve as starting points for efficient fine-tuning processes. In the low to medium *m*/*z* ranges, when fixing the *V*_s_ to the respective optimal value, the predicted extraction delays are only off by 0.6–13% from experimental observations. In our laboratory-made linear TOF mass spectrometer, under optimal ion focusing conditions, the *R*_m_ for the lower *m*/*z* range was enhanced 4.8–7.8 times with respect to the commercial instrument. At such a high level of *R*_m_, one must carefully consider factors like random errors in the instrument, the response time limit of the detector, and other practical limitations. After dynamic data correction minimizing random errors, the observed *R*_m_ values are around 25–40% less than predicted. These experimental results show that the coupling of space- and velocity-focusing is highly effective in pushing MALDI-TOF mass spectrometers to the extremes of their capacity. In order to further enhance the performance and extend mass range, new instrument design and electric systems need to be implemented. However, studies focusing on the contribution of other parameters are necessary to further improve the accuracy of prediction, such as the fringe effect of electric fields, the ion production and decay rates, etc. Extending the calculation model to reflectron or orthogonal extraction TOFMS is also attractive. These studies are currently under development and will be reported in the future.

## Supporting Information Available

Structure of the linear TOF mass spectrometer; comparison of flight time of ions with different *m*/*z* values; ion signal drift histogram; and the impact of extraction delay shift on the *R*_m_ (PDF)
