# Supplementary material for: Experimental Validation of Comprehensive Calculation for High-Resolution Linear MALDI-TOF Mass Spectrometry
Source: J Am Soc Mass Spectrom. 2024 Apr 18;35(5):992–8. doi: 10.1021/jasms.4c00018 (PMC11066958; doi:10.1021/jasms.4c00018)
Supplement: Supplementary file 1 — js4c00018_si_001.pdf [file js4c00018_si_001.pdf]

SUPPORTING INFORMATION

Experimental Validation of Comprehensive Calculation for High-Resolution Linear MALDI-TOF Mass Spectrometry

Yi-Hong Cai,[1] Chia-Chen Wang,[2] Chih-Hao Hsiao,[1] Yi-Sheng Wang[1]*

[1]Genomics Research Center, Academia Sinica, Taipei 115, Taiwan, ROC.

[2]Instrumentation Center, National Taiwan Normal University, Taipei 106, Taiwan, ROC

*To whom correspondence should be made, Email: wer@gate.sinica.edu.tw

Contents

Figure S1 The structure of linear TOF mass spectrometer. S2

Figure S2 Comparison between experimental results and computational predictions for the flight time of ions with different *m/z* values........ S2

Figure S3 The *m/z* 392 ion signal drift histogram. S3

Figure S4 The impact of extraction delay shift from the optimal condition on the R_m for the *m/z* 392 ion. S3

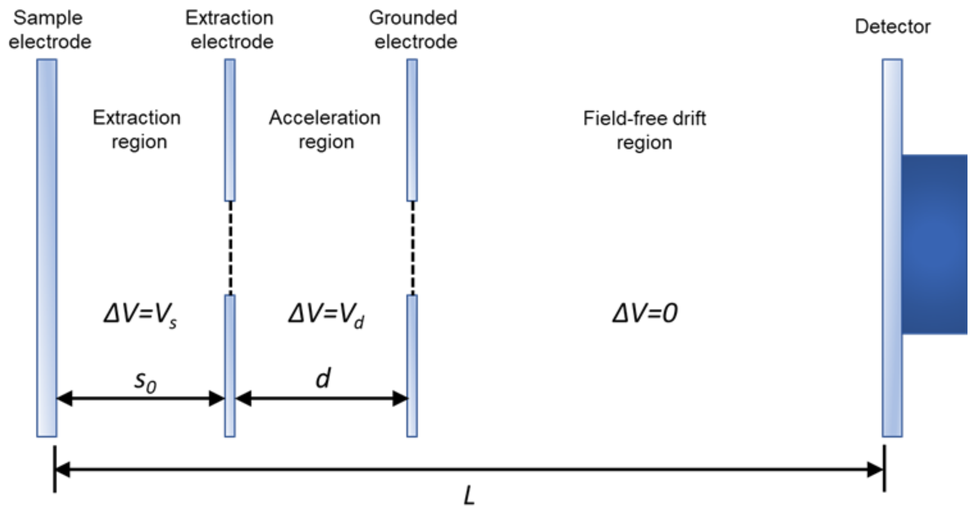

Figure S1 The structure of linear TOF mass spectrometer. This instrument includes two-stage extraction ion source encompasses an extraction and an acceleration region with lengths of 8 (s_0) and 10 (d) mm, respectively. The total length of the instrument (L) is 3,236 mm.

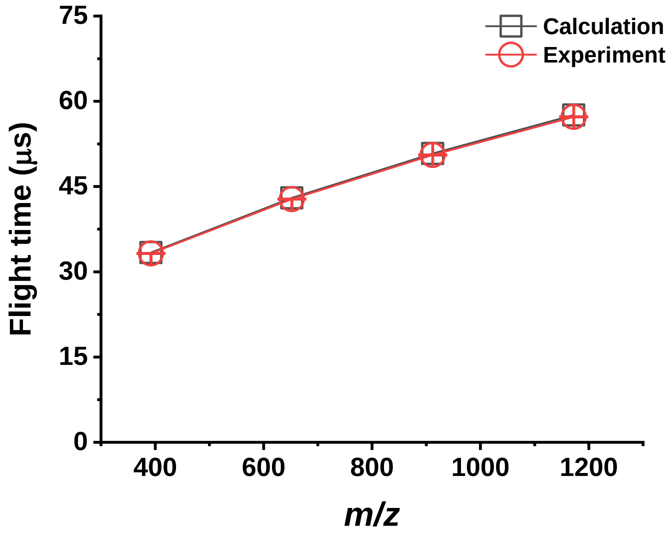

Figure S2 Comparison between experimental results and computational predictions for the flight time of ions with different *m/z* values. Instrument parameters are set as: total voltage at 20,000 V, s_0=8 mm, d=10 mm, and L=3,236 mm. The experimental parameters for various *m/z* ions are presented in Table 1. The results of the computational prediction are represented by the black line, and the experimental results are shown by the red line.

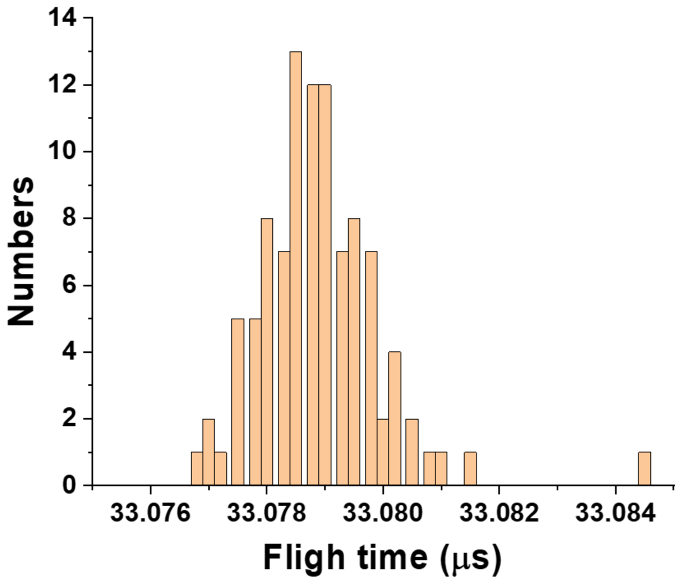

Figure S3 The *m/z* 392 ion signal drift histogram. The average flight time is approximately 33.079 μs with a standard deviation of about 1.06 ns. The ex-traction voltage and delay are respectively 930 V and 740 ns.

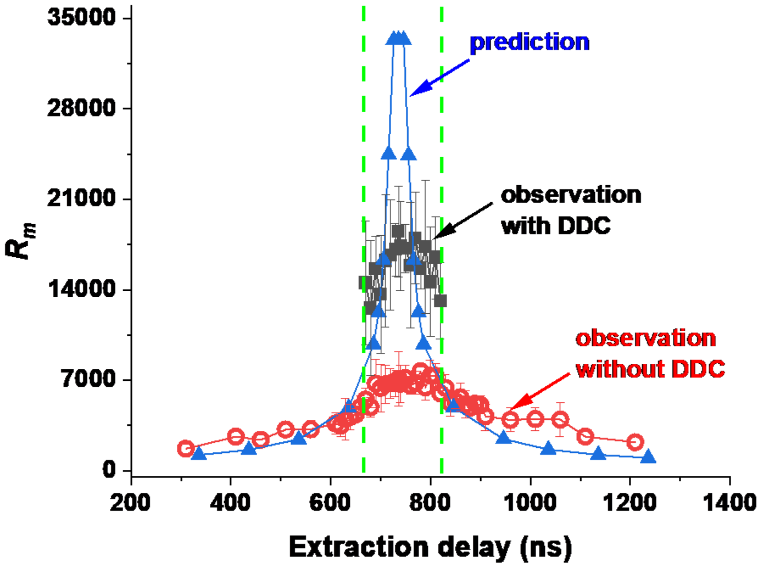

Figure S4 The impact of extraction delay shift from the optimal condition on the R_m for the *m/z* 392 ion. The instrument parameters are set as: total voltage at 20,000 V, s_0=8 mm, d=10 mm, V_s=930 V, and L=3,236 mm. Blue line: prediction; red line: experimental observation without correction; black: experimental observation with dynamic data correction.
